# Longitudinal Changes in Emergency Medical Services Advanced Airway Management

**DOI:** 10.1001/jamanetworkopen.2024.27763

**Published:** 2024-08-22

**Authors:** Henry E. Wang, Mengda Ivy Yu, Remle P. Crowe, Michelle M. J. Nassal, Christopher Gage, J. Madison Hyer, Jonathan Powell, Alexander Ulintz, Travis Sharkey-Toppen, Lai Wei, Kim Moeller, Ashish R. Panchal

**Affiliations:** 1 Department of Emergency Medicine, The Ohio State University, Columbus; 2 Center for Biostatistics, Department of Biomedical Informatics, The Ohio State University, Columbus; 3 ESO, Austin, Texas

## Abstract

**Importance:**

Identifying longitudinal changes in advanced airway management by emergency medical services (EMS) is crucial for understanding practice patterns and optimizing care.

**Objective:**

To examine the longitudinal trends in endotracheal intubation (ETI) and supraglottic airway (SGA) utilization in a national EMS cohort.

**Design, Setting, and Participants:**

This retrospective cross-sectional study analyzed 2011 to 2022 data from the ESO Data Collaborative, a national database of US prehospital electronic health records. The study included all 911 EMS events in which advanced airway management was attempted. Data were analyzed from November 2022 to January 2024.

**Exposures:**

Advanced airway management attempts, including ETI, SGA, and surgical airways.

**Main Outcomes and Measures:**

The annual percentage of ETI and SGA attempts, stratified by underlying condition (cardiac arrest, nonarrest medical, nonarrest trauma, pediatrics).

**Results:**

Among 47.5 million EMS activations, 444 041 (mean [SD] age, 60.6 [19.8] years; 273 296 [61.5%] men) involved advanced airway management, including 305 584 (68.8%) that used ETI and 200 437 (45.1%) that used SGA. The overall incidence was 9.3 per 1000 EMS events. In the cardiac arrest cohort from 2011 to 2022, EMS events with ETI attempts decreased from 2470 of 2831 (87.3%) to 40 083 of 72 793 (55.1%) and those with SGA attempts increased from 711 of 2831 (25.1%) to 44 386 of 72 793 (61.0%). In the pediatric subset, there were similarly large decreases in ETI attempts, from 117 of 182 EMS events (97.3%) to 1573 of 2307 EMS events (68.2%), and increases in SGA attempts, from 11 of 182 EMS events (6.6%) to 1058 of 2307 EMS events (45.9%). In the nonarrest medical and nonarrest trauma cohorts, ETI attempts decreased and SGA attempts increased but to a much lower extent.

**Conclusions and Relevance:**

In this national cross-sectional study of EMS care episodes, there were marked shifts in advanced airway management practices, with the increased use of SGA and decreased use of ETI. These observations highlight current trends in EMS airway management practices.

## Key Points

**Question:**

Have US Emergency Medical Services (EMS) endotracheal intubation (ETI) and supraglottic airway (SGA) utilization rates changed over time?

**Findings:**

In this cross-sectional study of 444 041 EMS activations in which patients underwent advanced airway management, there were marked increases in SGA attempts and corresponding decreases in ETI attempts, most prominently in the cardiac arrest and pediatric cohorts.

**Meaning:**

These findings highlight current trends in EMS advanced airway management practices.

## Teaser

This cross-sectional study examines the longitudinal trends in endotracheal intubation and supraglottic airway utilization for airway management in a national emergency medical services cohort.

## Introduction

Airway management is an important intervention in resuscitation from life-threatening conditions, such as cardiac arrest, trauma, and respiratory failure.^1^ As the first to provide life-saving resuscitation care, emergency medical services (EMS) personnel (paramedics and emergency medical technicians) often perform airway management on patients who are critically ill before they reach the hospital. Since its introduction to paramedic scope of practice more than 40 years ago, endotracheal intubation (ETI) has been the dominant advanced airway technique used in the prehospital setting in the US.^1^ However, numerous factors have influenced paramedic airway practices, including growing recognition of the drawbacks of ETI, knowledge generated by clinical trials, and clinical protocols implemented during the COVID-19 pandemic to mitigate rescuer exposure to pathogens while performing airway management.^1,2,3,4,5,6,7,8,9,10,11,12,13^ Over the last 10 years, there has been increasing EMS use of supraglottic airways (SGA), such as the laryngeal tube, laryngeal mask airway and i-gel (Intersurgical), in select prehospital patients who are critically ill, such as those experiencing cardiac arrest. Compared with ETI, SGA often entail a simpler and more rapid technique, have lower training burden, and offer similar ventilation characteristics.

Only limited data describe the current national patterns of EMS airway management in the US, including the relative proportions of ETI and SGA use.^14,15^ These insights are important for defining current EMS national clinical practices, identifying the number and proportions of patients subject to specific airway strategy, planning system deployment and configuration, and informing training strategies. These perspectives are also critical for receiving emergency department and intensive care unit teams, who must understand the technical nuances of different EMS airway techniques, the methods for transitioning or exchanging prehospital airway devices, and the strategies for managing associated complications. In this study, we sought to determine longitudinal trends in prehospital advanced airway management utilization in a national EMS cohort.

## Methods

### Study Design and Setting

This cross-sectional study was approved by the Ohio State University Office of Responsible Research Practices with a waiver of informed consent because of the secondary analysis of deidentified data. We followed the Strengthening the Reporting of Observational Studies in Epidemiology (STROBE) reporting guideline.

This cross-sectional study used EMS data from the ESO Data Collaborative. ESO is one of the largest EMS electronic health record systems in the US. The system records clinical information for EMS encounters, including event characteristics, patient demographics, clinical signs and symptoms, interventions, vital signs, and outcomes. Data validation (forcing) options are implemented in the software to require or constrain user entries for select data elements. The software follows National EMS Information System (NEMSIS) version 3 standards and associated data definitions.^11,12,13,16^ Currently, more than 2000 EMS agencies use the ESO electronic health record software system. The ESO Data Collaborative is a subset of records from EMS agencies that agree to provide deidentified data for research and benchmarking purposes. ESO data have been used in a range of research studies.^17,18,19,20^ We used data from the 2011 to 2022 ESO Data Collaborative.

### Selection of Participants

We identified all EMS patient encounters with attempts at advanced airway management, including ETI, SGA, and surgical airways. EMS personnel reported all airway management procedures attempted on each patient. ETI included orotracheal intubation, nasotracheal intubation, rapid-sequence intubation, sedation-assisted intubation, delayed-sequence intubation, retrograde intubation, and video laryngoscopy. SGA included laryngeal tubes, laryngeal mask airway, i-gel, esophageal-tracheal double lumen airway, and supraglottic airway laryngopharyngeal tube. Surgical airways included needle and surgical cricothyroidotomies and tracheostomies.

Because our objective was to characterize systemwide utilization of different airway management techniques, we included episodes with any attempts at each defined advanced airway procedure, regardless of the successful placement or number of attempts. Under this framework, an individual patient could undergo more than 1 advanced airway procedure. We also did not distinguish the use of adjuncts, such as the gum elastic bougie or video laryngoscopy.^21,22^

### Patient Subsets

EMS personnel indicated the presence of cardiac arrest using the cardiac arrest data element in the dataset, including arrests that occurred before or after EMS arrival. We did not use electrocardiographic data or resuscitation interventions (eg, chest compressions) to define cardiac arrest. EMS personnel also indicated the patient condition classification using the medical/trauma data element. We did not define trauma using injury patterns or severity. Given our focus on the patterns of attempted advanced airway management (and not clinical outcomes), if a patient was classified as both trauma and cardiac arrest, we classified the event as a cardiac arrest. Events related to neither cardiac arrest nor trauma were classified as nonarrest medical. We did not separately analyze other subsets, such as by use of drug assisted airway management or by supraglottic airway type. We separately defined the pediatric cohort as patients younger than 18 years.

### Statistical Analysis

We analyzed the data using descriptive statistics. We first determined the total and annual number of EMS activations during the study period, identifying longitudinal variations in baseline characteristics (age, sex, cardiac arrests, traumas) of the overall population. We excluded race and ethnicity from these presentations due to their lack of biological relevance to the study intervention.^23^ We then determined the number and annual incidence of advanced airway management events in the cohort. We determined the percentage of cases for each reported advanced airway technique. We determined the age, sex, cardiac arrest status, trauma status, and Census region of patients undergoing advanced airway management.

For each year, we determined the percentage of advanced airway events represented by cardiac arrests and patients with nonarrest medical events and nonarrest trauma events. For each year, we then determined the percentage of advanced airway events, including ETI and SGA attempts. We separately analyzed the cardiac arrest, nonarrest medical, and nonarrest trauma cohorts. We also separately analyzed the pediatric cohort. Because of the limited number of procedures, we excluded surgical airways from the longitudinal depiction.

We verified longitudinal trends of each airway type using unobserved components models (UCM), implemented using the SAS software (SAS Institute) command PROC UCM.^24^ In these models, the dependent variable was the percentage of ETI or SGA use, and the independent variable was the year (2011-2022). The UCM approach allowed us to identify temporal trends, which capture long-term movements. We did not include cyclical effects in our models, as our focus was on understanding the broader, long-term trends rather than shorter-term cycles. We used UCM to test for longitudinal changes over time due to its flexibility and robustness in handling complex time series data. We opted not to use other time series techniques, such as autoregressive integrated moving average and structural time series models because of their complexity and strong assumptions about underlying data structure. We used 2-sided *P* < .05 to indicate statistical significance.

To explore the potential effect of underlying population secular changes on the observed airway utilization trends, we repeated the analysis stratifying by key characteristics of the overall EMS response population, including age (≥65 vs <65 years), sex (male vs female), urbanicity (rural vs urban), and Census region (Midwest, Northeast, South, and West). We did not use multivariable longitudinal time series models to explore the confounding effect of population characteristics because the data were reported in 1-year intervals only. We conducted the analysis using SAS version 9.4 and Excel version 2405 (Microsoft). Data were analyzed from November 2022 to January 2024.

## Results

During 2011 to 2022, there were a total of 47.5 million EMS activations in the dataset (eTable 1 in Supplement 1). The annual number of EMS agencies contributing information to the dataset increased from 192 to 2670. Annual EMS events in the dataset increased from 547 627 to 9.7 million. Mean age, the percentage male sex, and clinical condition (cardiac arrest, medical, or trauma) remained relatively stable across the study period. The percentage of rural EMS events in the dataset declined from 19.2% in 2011 to 14.8% in 2022. Geographic representation in the dataset shifted across the study period: the percentage of EMS events from the Midwest Census region increased from 13.3% to 24.1%, and the percentage of events in the South Census region decreased from 63.9% to 47.1%.

During the study period, there was a total of 444 041 EMS activations (mean [SD] age, 60.6 [19.8] years; 273 296 [61.5%] men) with advanced airway management attempts (eTable 2 in Supplement 1). ETI accounted for 68.8% of advanced airway encounters; most airway management attempts were orotracheal intubation (Table). SGA accounted for 45.1% of advanced airway encounters. Surgical airway attempts accounted for only 0.2% of patient encounters.

**Table.** Methods Used for Advanced Airway Management

Method	Attempts, No. (%)^a^
Endotracheal intubation	305 584 (68.8)
Orotracheal intubation	244 352 (55.0)
Video laryngoscopy	39 126 (8.8)
Rapid sequence intubation	26 887 (6.1)
Nasotracheal intubation	6439 (1.5)
Sedation assisted intubation	2386 (0.5)
Retrograde intubation	494 (0.1)
Delayed sequence intubation	22 (<0.1)
Supraglottic airways	200 437 (45.1)
i-gel	102 007 (23.0)
Laryngeal tube	84 189 (19.0)
Laryngeal mask airway	9202 (2.1)
Esophageal tracheal double-lumen airway	5262 (1.2)
Other (supraglottic airway laryngopharyngeal tube)	224 (0.1)
Surgical airway	1000 (0.2)
Needle cricothyroidotomy	170 (<0.1)
Surgical cricothyroidotomy	811 (0.2)
Other techniques	121 (<0.1)

^a^ Percentages reflect portion of the 444 041 patients undergoing attempts with each airway method. Totals exceed 100% because an individual patient may have undergone more than one advanced airway technique.

During 2011 to 2022, the annual incidence of advanced airway management attempts ranged from 8.8 to 10.0 per 1000 EMS events (eFigure 1 in Supplement 1). Among patients undergoing advanced airway management, mean age and proportion male sex remained stable over the study period. The prevalence of rural advanced airway management events decreased from 22.3% to 16.9%. The Census region distribution of advanced airway cases shifted over the study period: events in the Midwest increased from 12.4% to 25.2%, events in the Northeast increased from 3.9% to 5.6%, events in the South decreased from 64.5% to 50.7%, and events in the West decreased from 19.1% to 14.7%.

Clinical condition categories associated with advanced airway management included cardiac arrest (343 312 events [77.3%]), nonarrest medical (79 358 events [17.9%]) and nonarrest trauma (21 371 events [4.8%]) (eFigure 2 in Supplement 1). The annual percentage of advanced airway attempts involving cardiac arrests increased from 54.9% to 82.2%. The annual percentage of advanced airway management attempts associated with nonarrest medical encounters decreased from 38.9% to 13.6%. The annual percentage of advanced airway management attempts associated with nonarrest trauma encounters decreased to a lesser extent (6.6% to 4.2%).

**Figure 1.**
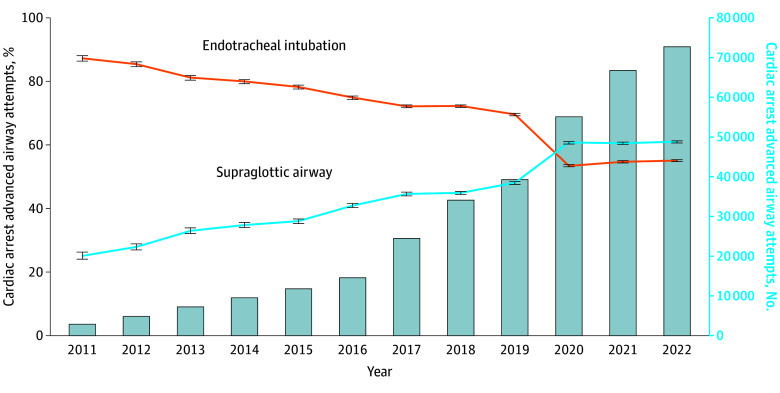
Longitudinal Trends in the Annual Percentage of Cardiac Arrest Endotracheal Intubation or Supraglottic Airway Insertion Attempts Data are from the 2011 to 2022 ESO Data Collaborative. Markers and whiskers indicate percentages and respective 95% CIs. Bar graph depicts total number of cardiac arrests undergoing advanced airway management attempts. Totals may exceed 100% because an individual patient may have undergone both supraglottic airway and endotracheal intubation attempts. Tests of trend from unobserved components models: endotracheal intubation *P* for trend < .001; supraglottic airway *P* for trend < .001.

**Figure 2.**
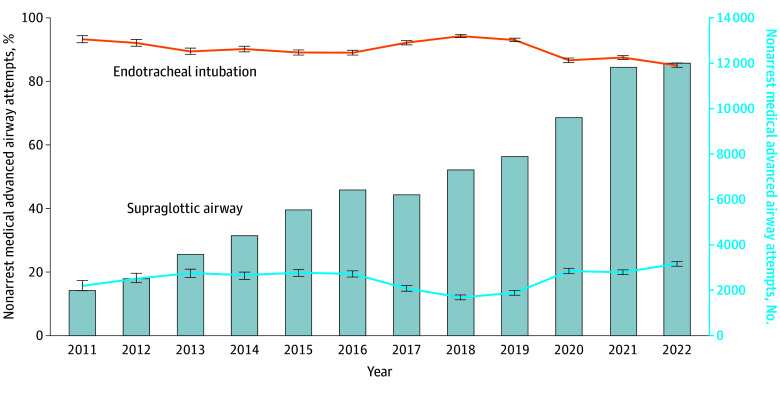
Longitudinal Trends in the Annual Percentage of Nonarrest Medical Endotracheal Intubation or Supraglottic Airway Insertion Attempts Data are from the 2011 to 2022 ESO Data Collaborative. Markers and whiskers indicate percentages and respective 95% CIs. Bar graph depicts total number of medical nonarrests undergoing advanced airway management attempts. Totals may exceed 100% because an individual patient may have undergone both supraglottic airway and endotracheal intubation attempts. Tests of trend from unobserved components models: endotracheal intubation *P* for trend = .33; supraglottic airway *P* for trend = .49.

**Figure 3.**
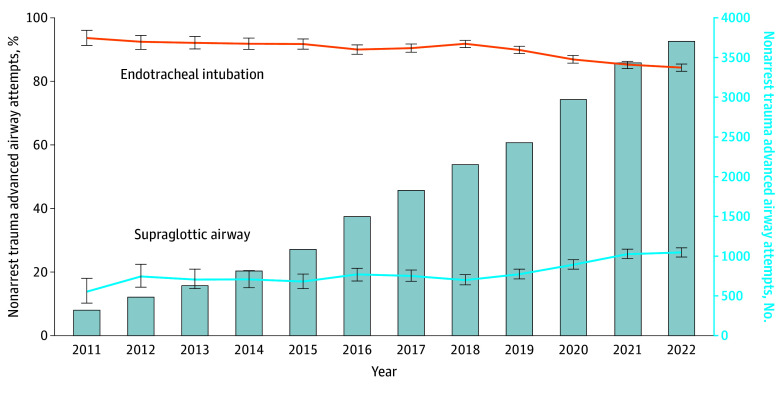
Longitudinal Trends in the Annual Percentage of Nonarrest Trauma Endotracheal Intubation or Supraglottic Airway Insertion Attempts Data are from the 2011 to 2022 ESO Data Collaborative. Markers and whiskers indicate percentages and respective 95% CIs. Bar graph depicts total number of trauma nonarrests with advanced airway management attempts. Totals may exceed 100% because an individual patient may have undergone both supraglottic airway and endotracheal intubation attempts. Tests of trend from unobserved components models: endotracheal intubation *P* for trend = .04; supraglottic airway *P* for trend = .07.

**Figure 4.**
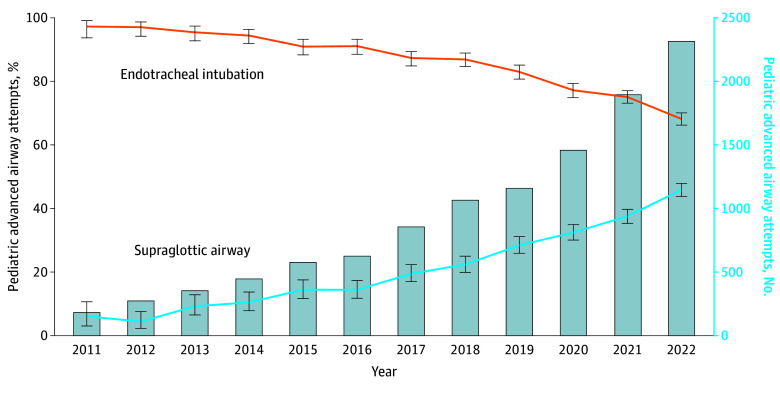
Longitudinal Trends in the Annual Percentage of Pediatric Endotracheal Intubation or Supraglottic Airway Insertion Attempts Data are from the 2011 to 2022 ESO Data Collaborative. Markers and whiskers indicate percentages and respective 95% CIs. Bar graph depicts total number of trauma nonarrests with advanced airway management attempts. Totals may exceed 100% because an individual patient may have undergone both supraglottic airway and endotracheal intubation attempts. Tests of trend from unobserved components models: endotracheal intubation *P* for trend = .002; supraglottic airway *P* for trend < .001.

In the cardiac arrest subset, ETI attempts decreased from 2470 of 2831 events (87.3%) to 40 083 of 72 793 events (55.1%), while SGA attempts increased from 711 of 2831 events (25.1%) to 44 386 of 72 793 events (61.0%) (Figure 1). Cardiac arrest SGA attempts exceeded ETI in 2020 to 2022. Changes in airway techniques for nonarrest medical events were less pronounced: ETI attempts decreased from 93.3% to 85.1%, while SGA attempts increased from 15.7% to 22.6% (Figure 2). There were similarly limited changes in advanced airway techniques for nonarrest trauma events: ETI decreased from 93.7% to 84.3% and SGA increased from 13.8% to 26.2% (Figure 3). In the pediatric subset, ETI attempts decreased from 117 of 182 events (97.3%) to 1573 of 2307 events (68.2%) while SGA attempts increased from 11 of 182 events (6.0%) to 1058 of 2307 events (45.9%) (Figure 4).

We focused the sensitivity analyses on the cardiac arrest and pediatric cohorts because of their visually distinct utilization patterns (eFigure 3 and eFigure 4 in Supplement 1). The longitudinal patterns of ETI and SGA use across patient age, sex, population setting, and Census region revealed consistent alignment over time, suggesting stable underlying demographic characteristics. While we observed some small longitudinal changes, these changes were minor and would not explain the observed overall trends in ETI and SGA proportions.

## Discussion

Our cross-sectional study offers important contemporary perspectives of the current patterns of EMS advanced airway management in the US. Using the large ESO dataset, which encompasses EMS events from across the US, we observed marked decreases in ETI attempts with contrasting increases in SGA attempts. These trends were most prominent in the cardiac arrest and pediatric cohorts. Our study is one of the few longitudinal descriptions of prehospital advanced airway management practices in the US and adds to evolving knowledge regarding EMS airway practices.

Our observations potentially reflect several influences on current EMS airway management practices. The role of paramedic ETI in clinical care is the source of intense international debate, with numerous studies highlighting the associated risks, including ET tube misplacement and dislodgment, oxygen desaturation and bradycardia, interference with cardiopulmonary chest compression quality, and iatrogenic hyperventilation.^1,5,6,11^ Attaining and maintaining ETI skills are additional challenges faced by paramedics.^1,8,9^ In 2018, results from the Pragmatic Airway Resuscitation Trial, the Airways-2 trial, and the Cardiac Arrest Airway Management trial—the first clinical trials of advanced airway management in adult out-of-hospital cardiac arrest (OHCA)—led to the recommended use of SGA in adult OHCA in settings with low ETI success rates or minimal ETI training opportunities.^10,11,12,13,25^ Before and after these trials, many EMS agencies implemented early use of SGAs to expedite care, optimize CPR chest compression quality, and mitigate delays and adverse events associated with ETI.^13,26^ A second important influence was the COVID-19 pandemic, when many EMS systems preferentially used SGAs instead of ETI to minimize exposure to infectious pathogens.^27,28^ The changes observed in the advanced airway management of children may be due to the broader availability of SGA in pediatric sizes and the parallel influence of adult airway practices. The alignment of our dataset with these multiple demographic characteristics over time further supports the robustness of our findings and minimizes the likelihood that such changes are artifacts of the data collection process.

The more modest shifts in ETI and SGA use in the nonarrest medical and trauma cohorts are not surprising. The rapidity of advanced airway insertion may be less urgent for medical and trauma nonarrests, allowing time for performing the more complex ETI. Given practice changes favoring SGA in cardiac arrests, some EMS medical directors have promoted ETI use in medical nonarrests and patients with trauma injuries to maintain opportunities for maintaining ETI procedural experience.^29^ The observed reductions in the overall proportions of nonarrest medical and trauma advanced airway management events may also signify the increasing use of bag-valve-mask ventilation rather than advanced airway management in these patients.

Clinicians in out-of-hospital and in-hospital settings should be aware of the trends illustrated in this study. When defining advanced airway management protocols, EMS medical directors must consider many factors, including an EMS agency’s patient mix, training opportunities and resources, and available airway equipment and adjuncts. Reports of current national practices will undoubtedly influence these individual EMS agency decisions. Since SGA devices are not designed for prolonged mechanical ventilation, hospital clinicians in emergency department, operating room, and intensive care unit settings must be familiar with their technical nuances and optimal methods for use. For example, a properly functioning SGA may be appropriate for initial resuscitation and assessment. However, since SGA may be associated with significant hypopharyngeal swelling, complicating direct laryngoscopy and intubation, some authorities recommend exchange with the assistance of a fiberoptic bronchoscope.^30,31^ The relative rates of aspiration between prehospital ETI and SGA are unknown but could influence subsequent ventilator management.

Using 2013 to 2022 data from NEMSIS, Gage et al^15^ similarly assessed longitudinal trends in the advanced airway management of more than 1 million OHCA. While there were declines in ETI use (from 73% to 63%) and increases in SGA use (from 27% to 37%), the changes were more modest than in our current ESO analysis. There are important contrasts between the 2 analyses. While large in scale (>25 million EMS events annually), NEMSIS combines data from multiple electronic health record system. All data from the ESO Data Collaborative originate from a single electronic health record system, mitigating variations in data assimilation. While Gage et al^15^ identified OHCA through NEMSIS indicators of chest compression or defibrillation, we identified cardiac arrest through paramedic indication of OHCA. We also included information on trauma, nonarrest medical, and pediatric encounters, offering important contrasting insights of these subsets.

### Limitations

This study has some limitations. These findings are ecologic in nature and cannot demonstrate causality. Unmeasured factors may have influenced the observed practice patterns. For example, the total number of EMS events and advanced airway management attempts in the dataset increased 14-fold over the 12-year study period; this is likely due to the increased number of EMS agencies represented in the dataset during this time. The mix of EMS agencies and personnel represented in the ESO Data Collaborative may have shifted during this time, with select agencies entering and exiting the dataset over time. The ESO system also transitioned from the NEMSIS version 2 to the version 3 standard in 2017.^32^ We did not adjust for confounders; we felt that these techniques would only cloud our central goal of characterizing the current clinical patterns of current EMS airway practices. However, we note that the characteristics of the overall EMS population remained relatively stable over the study period, and stratified analyses did not reveal any marked differences in the distribution of airway types, mitigating concern that the observations may reflect secular trends in the underlying population.

It is unknown whether the ESO Data Collaborative is nationally representative. There are approximately 23 000 EMS agencies in the US.^33^ The dataset used in this study encompasses more than 2000 EMS agencies that use the ESO electronic health system and consent to its use in the research dataset. The descriptions of airway management are based on EMS personnel self-reports and are not independently validated. However, the ESO dataset is national in scale, encompasses agencies from 50 US states or territories, and offers one of the largest and most detailed descriptions of current EMS care.

Because bag-valve-mask ventilation was reported inconsistently, we omitted the portion of patients who may have undergone bag-valve-mask ventilation only. We presented observations for ETI and SGA but not their subtypes. We characterized airway management attempts but not the success of complications of these efforts. We did not study procedural or patient outcomes. EMS personnel may have underreported the use of advanced airway management; the extent of this missingness is unknown. We studied systemwide patterns of advanced airway management patterns but not individual practitioner experience or proficiency. We did not study geographic units smaller than Census region. Our study did not evaluate the perspectives or beliefs of EMS practitioners, which may have driven advanced airway practices.

## Conclusions

In this national cross-sectional study of EMS care episodes, there were marked shifts in advanced airway management practices, with the increased use of SGA and decreased use of ETI. These observations highlight current trends in EMS airway management practices.

## Notes

Supplement 1. **eTable 1.** Longitudinal Characteristics of EMS Events in the ESO Data Collaborative, 2011-2022 **eTable 2.** Longitudinal Characteristics of EMS Events With Advanced Airway Management Attempts, ESO Data Collaborative, 2011-2022 **eFigure 1.** Longitudinal Trends in the Incidence of EMS Events With Advanced Airway Management Efforts **eFigure 2.** Longitudinal Trends in Annual Advanced Airway Management Cases, Stratified by Subgroups **eFigure 3.** Sensitivity Analysis of Longitudinal Trends in the Annual Percentage of Cardiac Arrests Undergoing Endotracheal Intubation or Supraglottic Airway Insertion Attempts, Stratified by Key Population Subsets **eFigure 4.** Sensitivity Analysis of Longitudinal Trends in the Annual Percentage of Pediatric Patients Undergoing Endotracheal Intubation or Supraglottic Airway Insertion Attempts, Stratified by Key Population Subsets

Supplement 2.
Data Sharing Statement
