# Supplementary material for: Longitudinal Changes in Emergency Medical Services Advanced Airway Management
Source: JAMA Netw Open. 2024 Aug 22;7(8):e2427763. doi: 10.1001/jamanetworkopen.2024.27763 (PMC11342135; doi:10.1001/jamanetworkopen.2024.27763)
Supplement: Supplement 1. — eTable 1. Longitudinal Characteristics of EMS Events in the ESO Data Collaborative, 2011-2022 eTable 2. Longitudinal Characteristics of EMS Events With Advanced Airway Management Attempts, ESO Data Collaborative, 2011-2022 eFigure 1. Longitudinal Trends in the Incidence of EMS Events With Advanced Airway Management Efforts eFigure 2. Longitudinal Trends in Annual Advanced Airway Management Cases, Stratified by Subgroups eFigure 3. Sensitivity Analysis of Longitudinal Trends in the Annual Percentage of Cardiac Arrests Undergoing Endotracheal Intubation or Supraglottic Airway Insertion Attempts, Stratified by Key Population Subsets eFigure 4. Sensitivity Analysis of Longitudinal Trends in the Annual Percentage of Pediatric Patients Undergoing Endotracheal Intubation or Supraglottic Airway Insertion Attempts, Stratified by Key Population Subsets [file jamanetwopen-e2427763-s001.pdf]

Supplemental Online Content

Wang HE, Yu MI, Crowe RP, et al. Longitudinal changes in emergency medical services advanced airway management. *JAMA Netw Open*. 2024;7(8):e2427763. doi:10.1001/jamanetworkopen.2024.27763

eTable 1. Longitudinal Characteristics of EMS Events in the ESO Data Collaborative, 2011-2022

eTable 2. Longitudinal Characteristics of EMS Events With Advanced Airway Management Attempts, ESO Data Collaborative, 2011-2022

eFigure 1. Longitudinal Trends in the Incidence of EMS Events With Advanced Airway Management Efforts

eFigure 2. Longitudinal Trends in Annual Advanced Airway Management Cases, Stratified by Subgroups

eFigure 3. Sensitivity Analysis of Longitudinal Trends in the Annual Percentage of Cardiac Arrests Undergoing Endotracheal Intubation or Supraglottic Airway Insertion Attempts, Stratified by Key Population Subsets

eFigure 4. Sensitivity Analysis of Longitudinal Trends in the Annual Percentage of Pediatric Patients Undergoing Endotracheal Intubation or Supraglottic Airway Insertion Attempts, Stratified by Key Population Subsets

This supplemental material has been provided by the authors to give readers additional information about their work.

© 2024 Wang HE et al. *JAMA Network Open.*

eTABLE 1

Longitudinal characteristics of EMS events in the ESO Data Collaborative, 2011-2022.

	YEAR												
**Characteristic**	**2011**	**2012**	**2013**	**2014**	**2015**	**2016**	**2017**	**2018**	**2019**	**2020**	**2021**	**2022**	**Total**
**EMS Agencies contributing to the data set - n**	192	268	376	500	641	784	1,135	1,1230	1,265	1,975	1,934	2,670	N/A
**EMS events – n**	547,627	850,889	1,220,935	1,608,091	2,045,966	2,420,342	3,510,724	4,539,266	5,648,080	7,242,258	8,206,021	9,695,552	47,535,751
**Age – Mean (SD)**	51.9 (24.7)	52.4 (24.5)	53.5 (24.4)	54.1 (24.3)	54.5 (24.2)	54.5 (24.1)	54.3 (24.0)	54.1 (23.8)	55.1 (23.8)	55.5 (23.3)	55.4 (23.4)	56.0 (23.7)	55.0 (23.8)
Missing	9,557	24,646	41,524	52,368	65,335	81,918	119,919	104,156	189,667	202,362	234,175	297,332	1,422,959
**Sex**													
Male – n (%)	246,841 (45.1%)	376,616 (44.3%)	538,677 (44.1%)	711,253 (44.2%)	910,620 (44.5%)	1,079,999 (44.6%)	1,575,751 (44.9%)	2,067,112 (45.5%)	2,593,575 (45.9%)	3,422,387 (47.3%)	3,846,646 (46.9%)	4,501,619 (46.4%)	21,871,096 (46.0%)
Female – n (%)	288,670 (52.7%)	446,545 (52.5%)	637,440 (52.2%)	839,092 (52.2%)	1,064,195 (52.0%)	1,247,699 (51.5%)	1,792,530 (51.1%)	2,299,314 (50.6%)	2,850,587 (50.5%)	3,558,756 (49.1%)	4,079,514 (49.7%)	4,872,293 (50.2%)	23,976,635 (50.4%)
Unknown – n (%)	0 (0.0%)	0 (0.0%)	0 (0.0%)	0 (0.0%)	0 (0.0%)	4 (<0.1%)	79 (<0.1%)	89 (<0.1%)	1,717 (<0.1%)	2,238 (<0.1%)	2,658 (<0.1%)	3,388 (<0.1%)	10,173 (<0.1%)
Not reported (missing) – n (%)	12,116 (2.2%)	27,728 (3.3%)	44,818 (3.7%)	57,746 (3.6%)	71,151 (3.5%)	92,640 (3.8%)	142,364 (4.1%)	172,751 (3.8%)	202,201 (3.6%)	258,877 (3.6%)	277,203 (3.4%)	318,252 (3.3%)	1,677,847 (3.5%)
**Urbanicity**													
Rural – n (%)	105,420 (19.2%)	163,053 (19.2%)	218,874 (17.9%)	297,983 (18.5%)	396,235 (19.4%)	467,304 (19.3%)	614,075 (17.5%)	715,545 (15.8%)	875,024 (15.5%)	1,095,286 (15.1%)	1,200,820 (14.6%)	1,439,059 (14.8%)	7,588,678 (16.0%)
Urban – n (%)	435,730 (79.6%)	677,402 (79.6%)	984,703 (80.7%)	1,277,536 (79.4%)	1,609,470 (78.7%)	1,921,980 (79.4%)	2,868,159 (81.7%)	3,786,955 (83.4%)	4,731,922 (83.8%)	6,088,066 (84.1%)	6,957,755 (84.8%)	8,193,850 (84.5%)	39,533,528 (83.0%%)
Missing – n (%)	6,477 (1.2%)	10,434 (1.2%)	17,358 (1.4%)	32,572 (2.0%)	40,261 (2.0%)	31,058 (1.3%)	28,490 (0.8%)	36,766 (0.8%)	41,134 (0.7%)	58,906 (0.8%)	47,446 (0.6%)	62,643 (0.7%)	413,545 (1.0%)
**Medical vs. Trauma**													
Medical – n (%)	265,389 (48.5%)	442,005 (52.0%)	653,877 (53.6%)	878,707 (54.6%)	1,148,695 (56.1%)	1,558,218 (64.4%)	2,478,773 (70.6%)	3,337,313 (73.5%)	4,160,406 (73.7%)	5,425,487 (74.9%)	6,164,291 (75.1%)	7,517,412 (77.5%)	34,030,573 (71.6%)
Trauma – n (%)	14,384 (2.6%)	21,999 (2.6%)	33,244 (2.7%)	44,506 (2.8%)	57,973 (2.8%)	66,305 (2.7%)	84,886 (2.4%)	109,342 (2.4%)	139,061 (2.5%)	182,327 (2.5%)	212,897 (2.6%)	260,410 (2.7%)	966,924 (2.0%)

© 2024 Wang HE et al. *JAMA Network Open.*

	YEAR												
**Characteristic**	**2011**	**2012**	**2013**	**2014**	**2015**	**2016**	**2017**	**2018**	**2019**	**2020**	**2021**	**2022**	**Total**
Medical and Trauma – n (%)	96,149 (17.6%)	144,420 (17.0%)	201,157 (16.5%)	261,983 (16.3%)	339,705 (16.6%)	434,934 (18.0%)	657,307 (18.7%)	867,940 (19.1%)	1,090,900 (19.3%)	1,300,303 (17.9%)	1,443,532 (17.6%)	1,689,746 (17.4%)	8,528,076 (17.9%)
Missing – n (%)	171,705 (31.4%)	242,465 (28.5%)	332,657 (27.2%)	422,895 (26.3%)	499,593 (24.4%)	360,885 (14.9%)	289,758 (8.2%)	224,671 (5.0%)	257,713 (4.6%)	334,141 (4.6%)	385,301 (4.7%)	488,394 (5.0%)	4,010,178 (8.4%)
**Cardiac Arrest**													
No – n (%)	542,863 (99.1%)	843,148 (99.1%)	1,208,883 (99.0%)	1,591,990 (99.0%)	2,026,222 (99.0%)	2,395,810 (99.0%)	3,469,454 (98.8%)	4,486,154 (98.8%)	5,580,052 (98.8%)	7,132,930 (98.5%)	8,091,224 (98.6%)	9,568,459 (98.7%)	46,937,189 (98.7%)
Yes – n (%)	4,764 (0.9%)	7,741 (0.9%)	12,052 (1.0%)	16,101 (1.0%)	19,744 (1.0%)	24,532 (1.0%)	41,270 (1.2%)	53,112 (1.2%)	68,028 (1.2%)	109,328 (1.5%)	114,797 (1.4%)	127,093 (1.3%)	598,562 (1.3%)
**Census Region**													
Midwest – n (%)	73,088 (13.3%)	125,993 (14.8%)	165,578 (13.6%)	294,851 (18.3%)	420,594 (20.6%)	511,649 (21.1%)	796,301 (22.7%)	1,004,859 (22.1%)	1,276,492 (22.6%)	1,779,476 (24.6%)	2,003,115 (24.4%)	2,336,692 (24.1%)	10,788,688 (22.7%)
Northeast – n (%)	31,301 (5.7%)	37,938 (4.5%)	59,945 (4.9%)	78,674 (4.9%)	113,900 (5.6%)	147,500 (6.1%)	147,466 (4.2%)	256,861 (5.7%)	270,721 (4.8%)	489,891 (6.8%)	542,795 (6.6%)	805,612 (8.3%)	2,982,604 (6.3%)
South – n (%)	350,095 (63.9%)	549,275 (64.5%)	808,251 (66.2%)	1,018,918 (63.4%)	1,240,680 (60.6%)	1,432,438 (59.2%)	2,080,570 (59.3%)	2,457,490 (54.1%)	3,129,953 (55.4%)	3,692,176 (51.0%)	4,155,268 (50.6%)	4,565,169 (47.1%)	25,480,283 (53.6%)
West – n (%)	92,950 (17.0%)	137,113 (16.1%)	185,728 (15.2%)	214,332 (13.3%)	268,523 (13.1%)	320,683 (13.2%)	476,416 (13.6%)	808,414 (17.8%)	933,089 (16.5%)	1,201,234 (16.6%)	1,409,516 (17.2%)	1,536,391 (15.8%)	7,584,389 (16.0%)
Missing – n (%)	193 (0.0%)	570 (0.1%)	1,433 (0.1%)	1,316 (0.1%)	2,269 (0.1%)	8,072 (0.3%)	9,971 (0.3%)	11,642 (0.3%)	37,825 (0.7%)	79,481 (1.1%)	95,327 (1.2%)	451,688 (4.7%)	699,787 (1.5%)

© 2024 Wang H et al. *JAMA Network Open.*

eTABLE 2

Longitudinal characteristics of EMS events with advanced airway management attempts, ESO Data Collaborative, 2011-2022.

	YEAR												
**Characteristic**	**2011**	**2012**	**2013**	**2014**	**2015**	**2016**	**2017**	**2018**	**2019**	**2020**	**2021**	**2022**	**Total**
**EMS events – n**	547,627	850,889	1,220,935	1,608,091	2,045,966	2,420,342	3,510,724	4,539,266	5,648,080	7,242,258	8,206,021	9,695,552	47,535,751
**Advanced Airway Management**	5,153	7,790	11,513	14,736	18,404	22,504	32,449	43,586	49,589	67,710	82,098	88,509	444,041
**Incidence (95% CI) - n per 1,000 EMS Events**	9.4	9.2	9.4	9.2	9.0	9.3	9.2	9.6	8.8	9.3	10.0	9.1	9.3
**Age – mean (SD)**	59.4 (20.7)	59.6 (20.5)	60.5 (20.2)	60.5 (20.2)	60.5 (20.3)	60.0 (20.1)	60.2 (20.0)	60.8 (19.7)	61.1 (19.5)	60.8 (19.6)	60.6 (19.6)	60.5 (19.9)	60.6 (19.8)
Missing	158 (3.1%)	219 (2.8%)	358 (3.1%)	388 (2.6%)	457 (2.5%)	588 (2.6%)	1,078 (3.3%)	1,619 (3.7%)	1,986 (4.0%)	2,974 (4.4%)	3,693 (4.5%)	122 (0.1%)	13,640 (3.1%)
**Sex**													
Male – n (%)	3,142 (61.0%)	4,629 (59.4%)	6,888 (59.8%)	8,833 (59.9%)	11,186 (60.8%)	13,755 (61.1%)	19,756 (60.9%)	26,771 (61.4%)	30,424 (61.4%)	41,946 (62.0%)	50,EMS (62.0%)	55,055 (62.2%)	273,296 (61.5%)
Female – n (%)	1,977 (38.4%)	3,095 (39.7%)	4,536 (39.4%)	5,804 (39.4%)	7,121 (38.7%)	8,607 (38.2%)	12,451 (38.4%)	16,455 (37.8%)	18,781 (37.9%)	25,381 (37.5%)	30,856 (37.6%)	33,228 (37.5%)	168,292 (37.9%)
Not reported – n (%)	34 (0.7%)	66 (0.8%)	89 (0.8%)	99 (0.7%)	97 (0.5%)	142 (0.6%)	241 (0.7%)	360 (0.8%)	377 (0.8%)	376 (0.6%)	327 (0.4%)	211 (0.2%)	2,419 (0.5%)
Unknown – n (%)	0 (0.0%)	0 (0.0%)	0 (0.0%)	0 (0.0%)	0 (0.0%)	0 (0.0%)	1 (0.0%)	0 (0.0%)	7 (0.0%)	7 (0.0%)	4 (0.0%)	15 (0.0%)	34 (0.0%)
**Urbanicity**													
Rural – n (%)	1,147 (22.3%)	1,704 (21.9%)	2,344 (20.4%)	3,040 (20.6%)	4,147 (22.5%)	5,160 (22.9%)	6,582 (20.3%)	7,960 (18.3%)	8,613 (17.4%)	11,644 (17.2%)	13,796 (16.8%)	14,924 (16.9%)	81,061 (18.3%)
Urban – n (%)	3,961 (76.9%)	6,006 (77.1%)	8,983 (78.0%)	11,333 (76.9%)	13,824 (75.1%)	17,037 (75.7%)	25,671 (79.1%)	35,308 (81.0%)	40,668 (82.0%)	55,635 (82.2%)	67,875 (82.7%)	73,096 (82.6%)	359,397 (80.9%)
Missing – n (%)	45 (0.9%)	80 (1.0%)	186 (1.6%)	363 (2.5%)	433 (2.4%)	307 (1.4%)	196 (0.6%)	318 (0.7%)	308 (0.6%)	431 (0.6%)	427 (0.5%)	489 (0.6%)	3,583 (0.8%)
**Medical vs. Trauma**													
Medical – n (%)	3,403 (66.0%)	5,471 (70.2%)	8,357 (72.6%)	10,811 (73.4%)	13,855 (75.3%)	18,968 (84.3%)	29,282 (90.2%)	39,794 (91.3%)	45,215 (91.2%)	61,861 (91.4%)	75,251 (91.7%)	81,041 (91.6%)	393,309 (88.6%)
Trauma – n (%)	419 (8.1%)	661 (8.5%)	864 (7.5%)	1,087 (7.4%)	1,466 (8.0%)	1,959 (8.7%)	2,893 (8.9%)	3,635 (8.3%)	4,245 (8.6%)	5,687 (8.4%)	6,651 (8.1%)	7,248 (8.2%)	36,815 (8.3%)
Missing – n (%)	1,331 (25.8%)	1,658 (21.3%)	2,292 (19.9%)	2,838 (19.3%)	3,083 (16.8%)	1,577 (7.0%)	274 (0.8%)	157 (0.4%)	129 (0.3%)	162 (0.2%)	196 (0.2%)	220 (0.2%)	13,917 (3.1%)

© 2024 Wang H et al. *JAMA Network Open.*

	YEAR												
Characteristic	2011	2012	2013	2014	2015	2016	2017	2018	2019	2020	2021	2022	Total
**Cardiac Arrest**													
Yes – n (%)	2,831 (54.9%)	4,785 (61.4%)	7,293 (63.4%)	9,510 (64.5%)	11,761 (63.9%)	14,582 (64.8%)	24,408 (75.2%)	34,125 (78.3%)	39,269 (79.2%)	55,131 (81.4%)	66,824 (81.4%)	72,793 (82.2%)	343,312 (77.3%)
No – n (%)	17 (0.3%)	30 (0.4%)	63 (0.6%)	79 (0.5%)	81 (0.4%)	84 (0.4%)	82 (0.2%)	95 (0.2%)	108 (0.2%)	150 (0.2%)	195 (0.2%)	214 (0.2%)	1,198 (0.3%)
Missing – n (%)	2,305 (44.7%)	2,975 (38.2%)	4,157 (36.1%)	5,147 (34.9%)	6,562 (35.7%)	7,838 (34.8%)	7,959 (24.5%)	9,366 (21.5%)	10,212 (20.6%)	12,429 (18.4%)	15,079 (18.4%)	15,502 (17.5%)	99,531 (22.4%)
**Census Region**													
Midwest – n (%)	638 (12.4%)	1,116 (14.3%)	1,507 (13.1%)	2,734 (18.6%)	3,950 (21.5%)	5,321 (23.6%)	7,547 (23.3%)	8,657 (19.9%)	11,284 (22.8%)	16,392 (24.2%)	20,422 (24.9%)	22,266 (25.2%)	101,834 (22.9%)
Northeast – n (%)	201 (3.9%)	218 (2.8%)	361 (3.1%)	461 (3.1%)	640 (3.5%)	758 (3.4%)	947 (2.9%)	1,607 (3.7%)	1,876 (3.8%)	2,950 (4.4%)	3,875 (4.7%)	4,924 (5.6%)	18,818 (4.2%)
South – n (%)	3,326 (64.5%)	5,014 (64.4%)	7,648 (66.4%)	9,438 (64.0%)	11,392 (61.9%)	13,581 (60.4%)	19,653 (60.6%)	21,904 (50.2%)	28,818 (58.1%)	38,394 (56.7%)	45,126 (55.0%)	44,885 (50.7%)	249,179 (56.1%)
West – n (%)	984 (19.1%)	1,437 (18.4%)	1,981 (17.2%)	2,087 (14.2%)	2,403 (13.1%)	2,822 (12.5%)	4,281 (13.2%)	5,342 (12.3%)	7,155 (14.4%)	9,563 (14.1%)	12,036 (14.7%)	13,005 (14.7%)	63,096 (14.2%)
Missing – n (%)	4 (0.1%)	5 (0.1%)	16 (0.1%)	16 (0.1%)	19 (0.1%)	22 (0.1%)	21 (0.1%)	6,076 (13.9%)	456 (0.9%)	411 (0.6%)	639 (0.8%)	3,429 (3.9%)	11,114 (2.5%)

© 2024 Wang H et al. *JAMA Network Open.*

eFIGURE 1

Longitudinal trends in the incidence of EMS events with advanced airway management efforts. Bar graph depicts total number of advanced airway cases in the data set. ESO Data Collaborative 2011-2022. Test of trend from unobserved components model: *p* for trend=0.62.

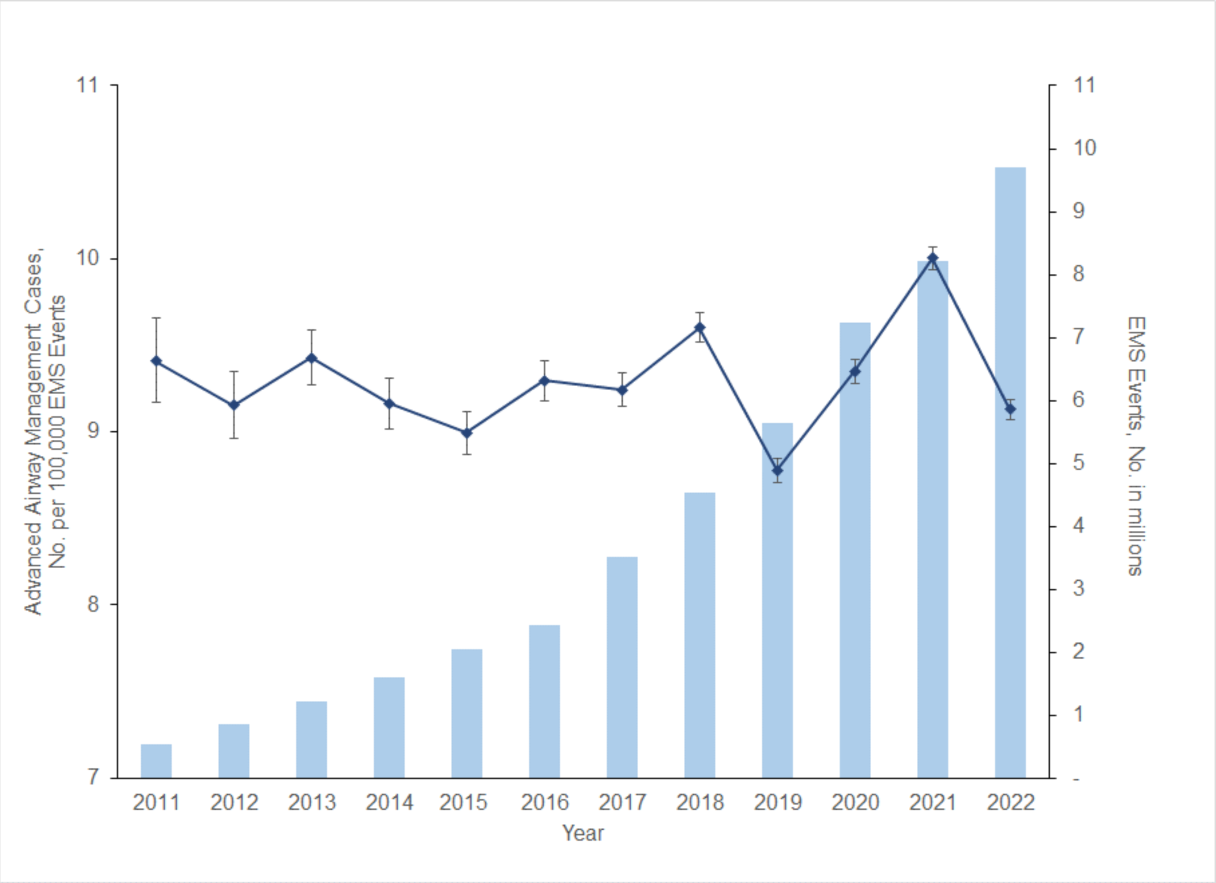

© 2024 Wang H et al. *JAMA Network Open.*

eFIGURE 2

Longitudinal trends in annual advanced airway management cases, stratified by subgroups (cardiac arrest, non-arrest medical and non-arrest trauma, and pediatrics). ESO Data Collaborative, 2011-2022. Bar graph depicts total number of advanced airway management cases in the data set. Includes 343,312 cardiac arrest, 79,358 non-arrest medical, and 21,371 non-arrest trauma and 11,204 pediatric advanced airway management cases. Tests of trend from unobserved components models: Cardiac arrest *p* for trend=0.002. Non-arrest medical *p* for trend<0.001. Non-arrest trauma *p* for trend<0.01. Pediatrics *p* for trend<0.001.

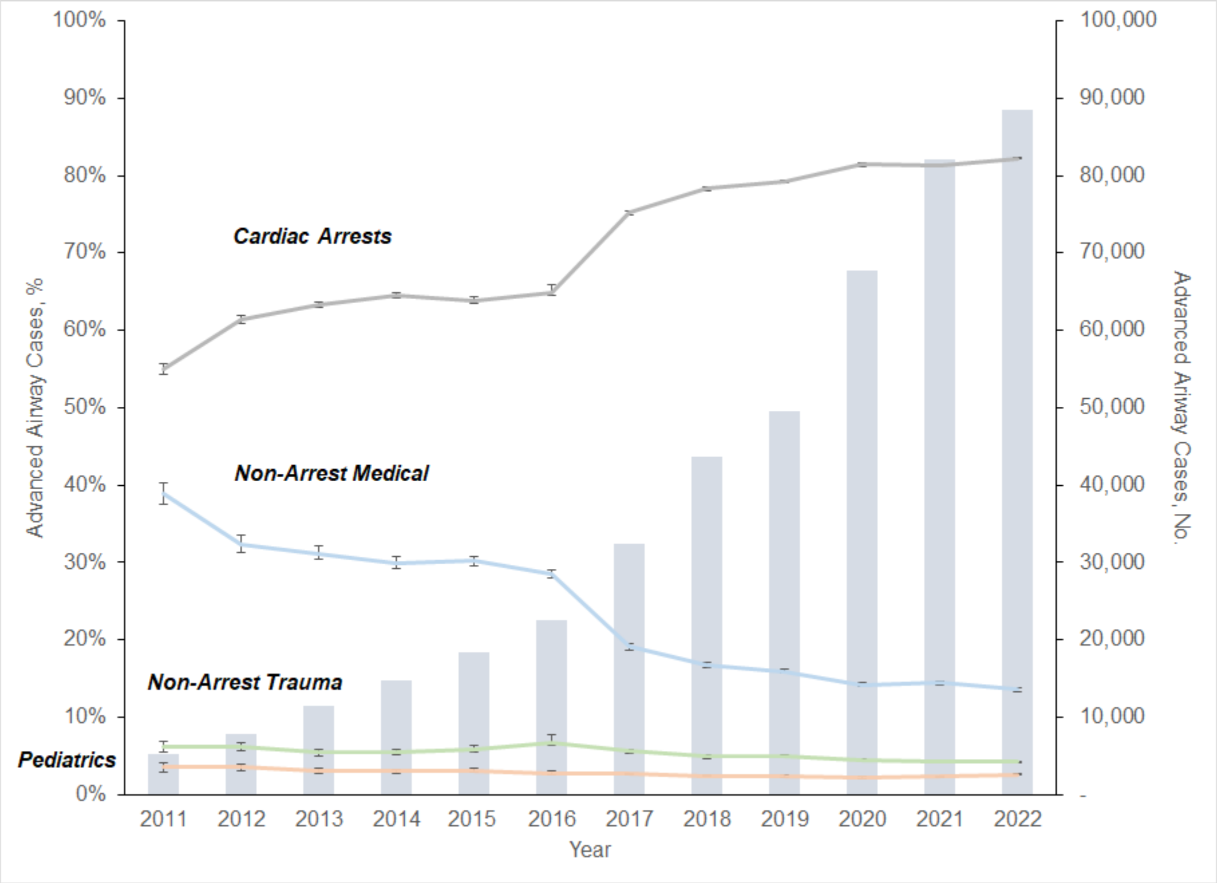

© 2024 Wang H et al. *JAMA Network Open.*

eFIGURE 3

Sensitivity analysis. Longitudinal trends in the annual percentage of cardiac arrests undergoing endotracheal intubation (ETI) or supraglottic airway (SGA) insertion attempts, stratified by key population subsets (patient age, sex, population setting, census region). ESO Data Collaborative, 2011-2022. Totals may exceed 100% because an individual patient may have undergone both SGA and ETI attempts.

Age<65 years

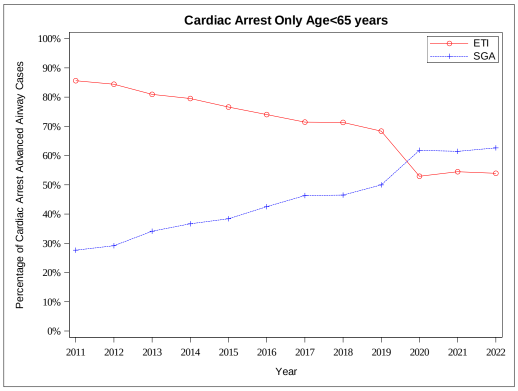

Age≥65 years

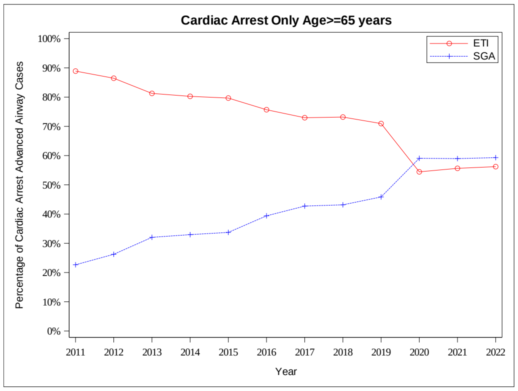

Male Sex

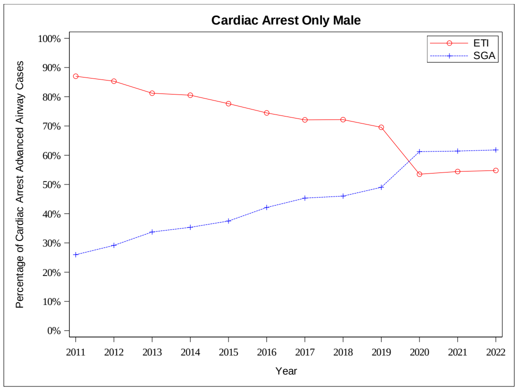

Female Sex

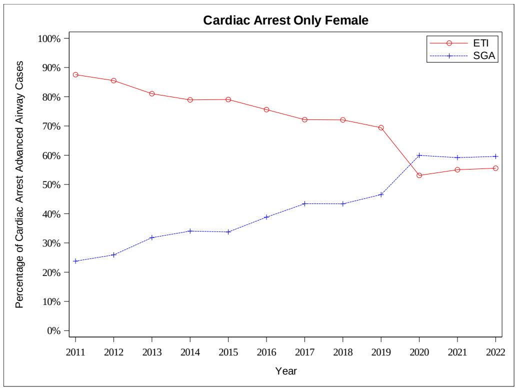

Urban Population Setting

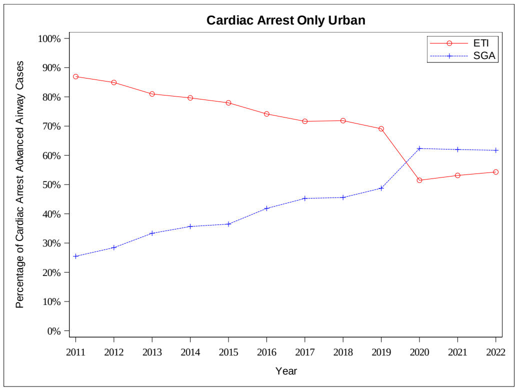

Rural Population Setting

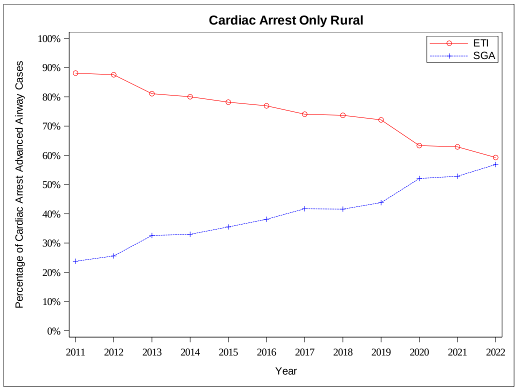

© 2024 Wang HE et al. *JAMA Network Open.*

Northeast Census Region

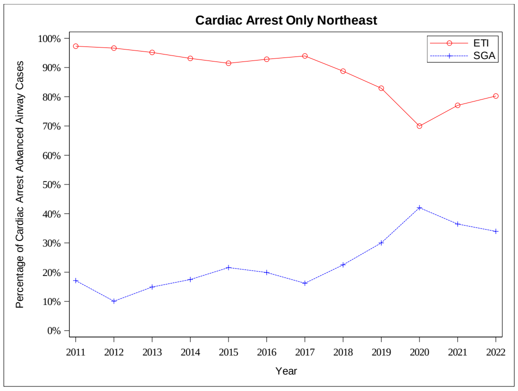

Midwest Census Region

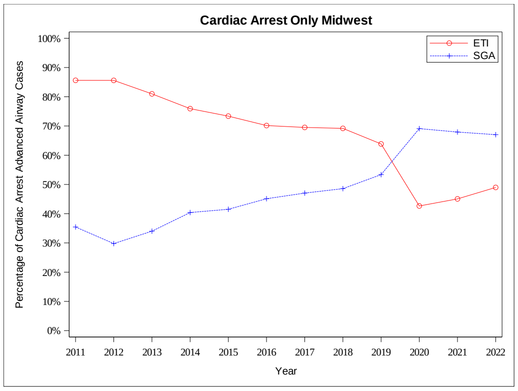

South Census Region

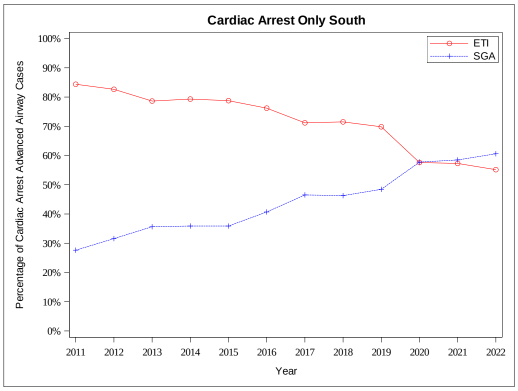

West Census Region

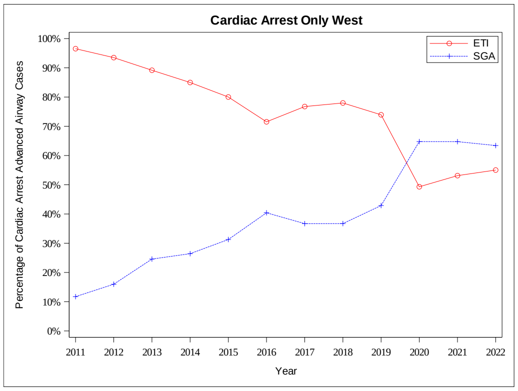

© 2024 Wang H et al. *JAMA Network Open.*

eFIGURE 4

Sensitivity analysis. Longitudinal trends in the annual percentage of pediatric patients undergoing endotracheal intubation (ETI) or supraglottic airway (SGA) insertion attempts, stratified by key population subsets (patient sex, population setting, census region). ESO Data Collaborative, 2011-2022. Totals may exceed 100% because an individual patient may have undergone both SGA and ETI attempts.

Male Sex

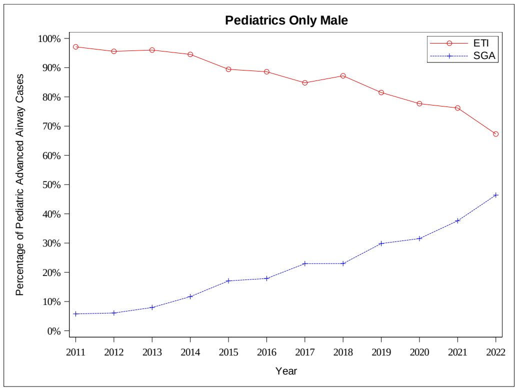

Female Sex

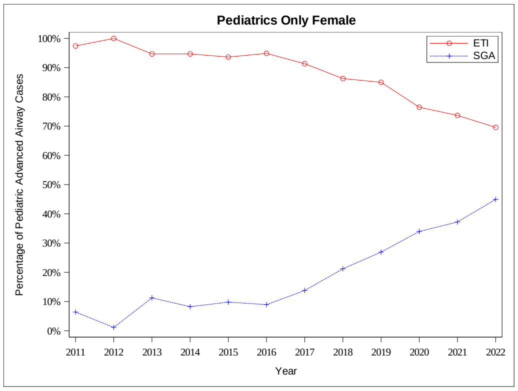

Urban Population Setting

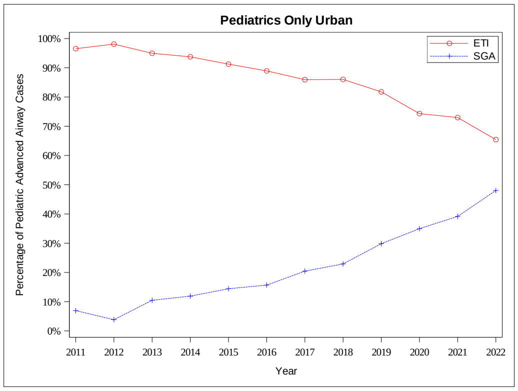

Rural Population Setting

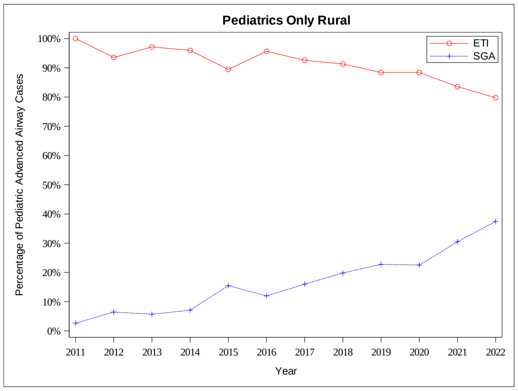

© 2024 Wang H et al. *JAMA Network Open.*

Northeast Census Region

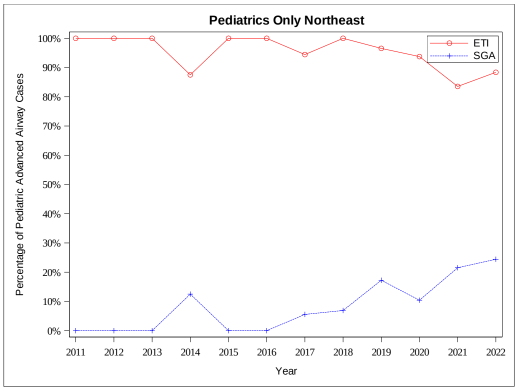

Midwest Census Region

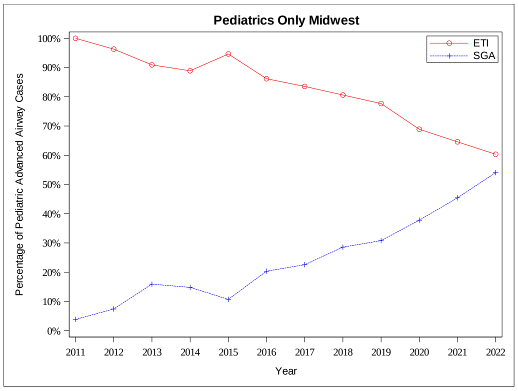

South Census Region

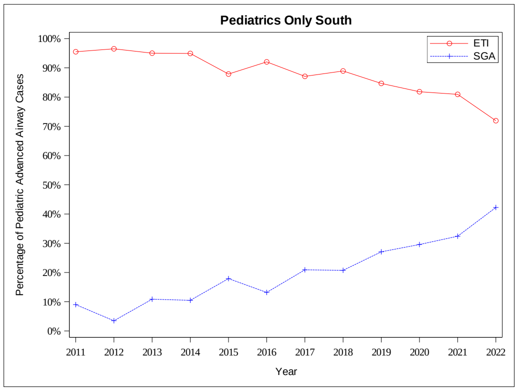

West Census Region

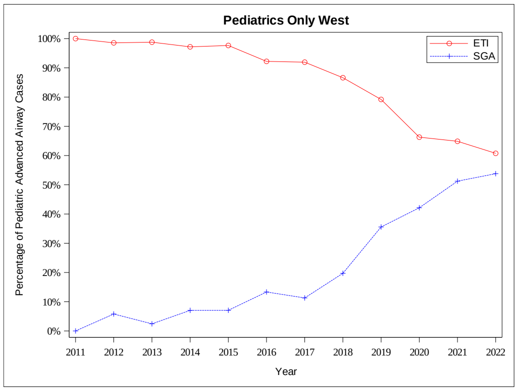

© 2024 Wang H et al. *JAMA Network Open.*
