# Supplementary material for: Longitudinal Changes in Emergency Medical Services Advanced Airway Management
Source: JAMA Netw Open. 2024 Aug 22;7(8):e2427763. doi: 10.1001/jamanetworkopen.2024.27763 (PMC11342135; doi:10.1001/jamanetworkopen.2024.27763)
Supplement: Supplement 2. — Data Sharing Statement [file jamanetwopen-e2427763-s002.pdf]

Data Sharing Statement

Wang. Longitudinal Changes in Emergency Medical Services Advanced Airway Management. *JAMA Netw Open*. Published August 22, 2024. doi:10.1001/jamanetworkopen.2024.27763

Data
Data available: Yes
Data types: Deidentified participant data
How to access data: The data used in this study may be requested from ESO, Inc., Austin. Texas
When available: With publication

Supporting Documents
Document types: None

Additional Information
Who can access the data: Anyone requesting the data
Types of analyses: For any purpose
Mechanisms of data availability: The data used in this study may be requested from ESO, Inc., Austin. Texas
